# RANKL Promotes Migration and Invasion of Hepatocellular Carcinoma Cells via NF-κB-Mediated Epithelial-Mesenchymal Transition

**DOI:** 10.1371/journal.pone.0108507

**Published:** 2014-09-30

**Authors:** Fang-Nan Song, Meng Duan, Long-Zi Liu, Zhi-Chao Wang, Jie-Yi Shi, Liu-Xiao Yang, Jian Zhou, Jia Fan, Qiang Gao, Xiao-Ying Wang

**Affiliations:** 1 Liver Cancer Institute, Zhongshan Hospital, and Key Laboratory of Carcinogenesis and Cancer Invasion (Ministry of Education), Fudan University, Shanghai, P. R. China; 2 Institute of Biomedical Sciences, Fudan University, Shanghai, P. R. China; The University of Hong Kong, China

## Abstract

**Background:**

Metastasis accounts for the most deaths in patients with hepatocellular carcinoma (HCC). Receptor activator of nuclear factor kappa B ligand (RANKL) is associated with cancer metastasis, while its role in HCC remains largely unknown.

**Methods:**

Immunohistochemistry was performed to determine the expression of RANK in HCC tissue (n = 398). Quantitative real-time polymerase chain reaction (qRT-PCR) and Western blot were used to examine the expression of RANK, E-cadherin, N-cadherin, vimentin, Snail, Slug, Twist and MMPs in HCC cells. Wound healing and Transwell assays were used to evaluate cell migration and invasion ability.

**Results:**

We found that expression of RANK, the receptor of RANKL, was significantly higher in HCC tumor tissues than in peritumor liver tissues (*p*<0.001). Constitutive expression of RANK was detected in HCC cell lines, which can be up-regulated when HCC cells were stimulated with RANKL. Notably, *in vitro* experiments showed that activation of RANKL-RANK axis significantly promoted migration and invasion ability of HCC cells. In addition, RANKL stimulation increased the expression levels of N-cadherin, Snail, and Twist, while decreased the expression of E-cadherin, with concomitant activation of NF-κB signaling pathway. Moreover, administration of the NF-κB inhibitor attenuated RANKL-induced migration, invasion and epithelial-mesenchymal transition of HCC cells.

**Conclusions:**

RANKL could potentiate migration and invasion ability of RANK-positive HCC cells through NF-κB pathway-mediated epithelial-mesenchymal transition, which means that RANKL-RANK axis could be a potential target for HCC therapy.

## Introduction

Hepatocellular carcinoma (HCC) is the fifth most common cancer and the third leading cause of death by cancer, with more than half a million new cases worldwide each year [1], [2]. Intrahepatic and extrahepatic metastasis is frequently the fatal step in the progression of HCC [3]. A key feature of cancer metastasis is enhanced motility and invasive behavior of the tumor cells [4].The increased motility, invasive ability and metastasis of tumor cells are widely associated with epithelial-mesenchymal transition (EMT), which also plays key roles in normal physiological processes such as embryogenesis, wound repair, and tissue remodeling [5], [6], [7]. EMT is a development process whereby epithelial cells undergo dramatic morphology changes, characterized by loss of their polarity and cell-cell contacts [5], [8]. The molecular hallmarks for EMT are down-regulation of epithelial markers (e.g., E-cadherin), and up-regulation of mesenchymal markers (e.g., vimentin and N-cadherin) [5], [9]. The induction of EMT can be triggered by transcription factors such as Snail, Slug, and Twist, which simultaneously repress the expression of genes that are required for the epithelial phenotype and induce the expression of genes required for mesenchymal properties [10]. The expression of these transcription factors is modulated by a number of signaling molecules, including nuclear factor kappa B (NF-κB) [10], [11]. NF-κB is a dimeric transcription factor composed of members of the Rel family, including RelA (p65) [12], [13]. Although nuclear translocation of the dimers is necessary for activating expression of its target genes, posttranslational modifications such as p65 phosphorylation can potentiate NF-κB-mediated transcriptional activity [12], [14], [15].

Receptor activator of nuclear factor kappa B ligand (RANKL, also called TNFSF11) is a member of the tumor necrosis factor superfamily [16]and, after binding to its cognate receptor RANK (also called TNFSF11A) [17], is a potent stimulator of NF-κB [18]. The expression of both RANKL and RANK has been observed in diverse types of malignant human tumors, and their expression correlates with metastasis and poor patient survival [19]. RANK also played a key role in tumor cell migration and invasion [20], [21]. Moreover, it has been demonstrated that RANKL promotes migration, and invasion of several types of human tumor cells expressing its receptor RANK [22], [23], [24]. However, the role of RANKL-RANK axis in modulating the behaviors of HCC cells is mostly unknown. Here we showed that RANKL directly promoted migration, invasion, and EMT of RANK-positive HCC cells via the NF-κB pathway.

## Materials and Methods

### Patients and specimens

Archived specimens for tissue microarray construction were obtained from a cohort of 398 patients who received curative resection of HCC at liver Cancer Institute, Zhongshan Hospital of Fudan University in 2006 between January and November. Patients neither showed signs of distant metastasis nor had they received anticancer therapy before surgery. Ethical approval was obtained from the Zhongshan Hospital Research Ethics Committee and written informed consent was obtained from each participant. HCC tissue microarrays were constructed as described previously [25].

### Cell culture

HCC cell lines, Huh-7(Japanese Cancer Research Bank) and HepG2 (American Type Culture Collection) were cultured in high-glucose Dulbecco's modified Eagle medium (DMEM; Invitrogen) supplemented with 10% fetal bovine serum (FBS; Invitrogen), 100 U/ml penicilin, and 100 µg/ml streptomycin(Sigma-Aldrich) at 37°C in a 5% CO_2_ humidified incubator. Human soluble RANKL was purchased from PeproTech (NJ, USA). Helenalin, a specific NF-κB inhibitor, was obtained from Santa Cruz Biotechnology (CA, USA). For experiments, cells were incubated with 100 ng/ml RANKL or the appropriate negative control (phosphate buffer solution; PBS) for different periods of time before which some were pretreated with1 µM helenalin or PBS for 60 minutes according to previously published works [22], [26].

### Immunohistochemistry

Immunohistochemistry was performed according to a previously described two-step protocol (Novocastra, Newcastle, UK).The primary antibody used was mouse monoclonal anti-human RANK antibody (Abcam, UK) [27]. Briefly, paraffin sections were deparaffinized, and the endogenous peroxidase was neutralized with 0.3% H_2_O_2_. After antigen retrieval was performed in a microwave oven in pH 6.0 citrate buffer, the sections were incubated with primary and secondary antibodies. The tissue microarray was then stained with diaminobenzidine (DAB) and counterstained with hematoxylin. Each case was scored by two independent pathologists using semi-quantitative method, evaluating the heterogeneous positive distribution and the differing intensity of the staining simultaneously. The staining score was divided into four grades: −, negative; +, mild; ++, intermediate; and +++, strong as described previously [28].

### Quantitative RT-PCR

Total RNA was isolated using TRIzol reagent (Invitrogen, Carlsbad, CA, USA) and treated with DNase to avoid amplification of genomic DNA. One microgram of total mRNA was reverse transcribed with oligo (dT) primers into complementary DNA (cDNA) using SuperScript III Reverse Transcriptase (Invitrogen). Quantitative real-time PCR was performed on a ABI Prism 7900 Sequence Detection System (Applied Biosystems, Foster City, CA, USA) starting with 1 µl cDNA, sequence-specific primers, and SYBR Green Realtime PCR Master Mix (Takara, Japan) at the following thermal conditions: 95°C for 10 min and 40 cycles at 95°C for 15 s and 60°C for1 min. Primers used were listed in Table S1. Relative mRNA levels were calculated by the comparative cycle threshold (Ct) method (2^−ΔΔCt^ formula) [29] with glyceraldehyde 3-phosphate dehydrogenase (GAPDH) for normalization. All samples were performed in triplicate for three times.

### Western blot analysis

The whole-cell lysates were extracted with RIPA lysis buffer (Beyotime, China) containing phenylmethylsulfonyl fluoride (PMSF); the cytosol and nuclear extracts were prepared using NE-PER Nuclear and Cytoplasmic Extraction Reagents (Pierce Biotechnology). Protein samples were separated by sodium dodecyl sulfate-polyacrylamide gel electrophoresis (SDS-PAGE), and electrotransferred onto polyvinylidene difluoride (PVDF) membranes (Millipore, Bedford, MA, USA). The membranes were blocked with 5% nonfat milk, washed, and then probed with primary antibodies listed in Table S2. After washing, the membrane was incubated with horseradish peroxidase(HRP)-conjugated secondary antibodies (Santa Cruz Biotechnology) and detected using enhanced chemiluminescence method (Pierce, Rockford, IL, USA). The amounts of proteins were quantified by densitometry with ImageJ software (National Institutes of Health, Bethesda, MD, USA) and normalized to the relative internal standards. All of the experiments were performed in triplicate.

### Wound healing assay

Cells were seeded into 24-well flat-bottomed plates at a density of 1×10^5^ cells per well and allowed to grow to 90% confluence. After aspirating the medium, the monolayer was scratched with a sterile 10 µl pipette tip to create a denuded zone (gap) of constant width. The remaining cells were washed twice with PBS to remove cell debris and incubated at 37°C in serum-free medium alone or containing 100 ng/ml RANKL. The scratched areas were photographed at 0 and 72 hours after wounding using a phase-contrast microscopy (magnification ×200). Cell migration was calculated as percentages of cell coverage to the initial cell-free zone using ImageJ software. The values are the means of three independent experiments.

### Transwell invasion assay

Cell invasion assays were performed using 24-well Transwells (8-µm pore size; Minipore) precoated with 100 µl Matrigel (BD Biosciences, Franklin Lakes, NJ) overnight at room temperature. Cells in 100 µl DMEM containing 1% FBS were seeded in the upper chamber at a density of 1×10^5^ cells per well, and 600 µl DMEM containing 1% FBS in the presence or absence of 100 ng/ml RANKL was added to the lower chamber. After incubation at 37°C for 24 h, Matrigel and any remaining cells in the upper layer were removed by cotton swabs and penetrating cells in the lower layer were fixed in 4% paraformaldehyde and stained with Giemsa. For quantification, cells in 5 randomly selected microscopic fields (magnification ×200) were counted and photographed using inverted phase-contrast microscopy. All experiments were performed in triplicate.

### Statistical analysis

All data were analyzed with PASW software version 20 (IBM, USA). Data were presented as means ± standard deviation (S.D.). Comparisons between groups were performed using Chi-square test, Student's t-test, or One-way analysis of variance (ANOVA) when appropriate. P<0.05 (two-tailed) was considered to be significant.

## Results

### RANK was up-regulated in human HCC and RANKL promoted migration and invasion of HCC cells

**Figure 1:**
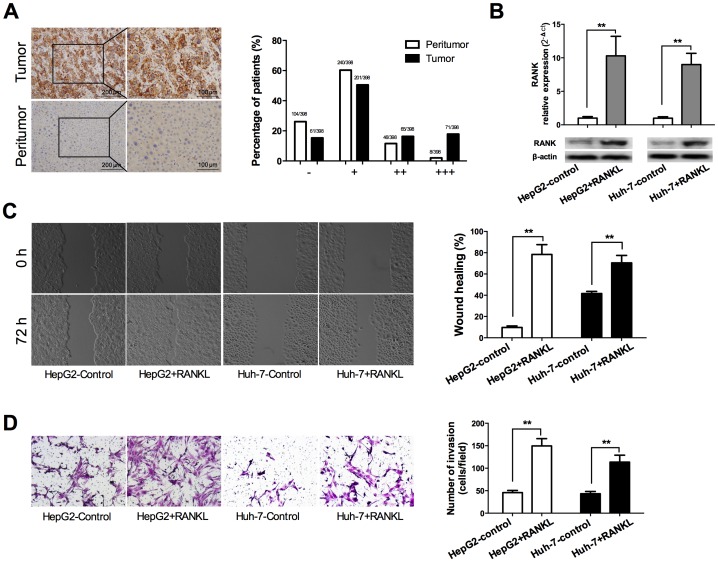
RANK was up-regulated in HCC and RANKL promoted migration and invasion of HCC cells. A. Left: Representative immunostaining images showed overexpression of RANK in HCC tumor tissues than paired peritumor tissues. Right: Bar graph showed the statistics of staining-intensity analysis in 398 HCC patients. RANK expression level in tumor tissues was significantly higher than that in peritumor tissues (*p*<0.001). B. HepG2 and Huh7 cells were incubated with 100 ng/ml RANKL or PBS as control for 24 h. qRT-PCR and Western blot analyses indicated that RANKL significantly promoted the expression of RANK. C. HepG2 and Huh7 cells were incubated with 100 ng/ml RANKL or PBS as control for 24 h. Wound healing assay demonstrated RANKL significantly promoted migration ability of the two cell lines (magnification ×200). D. HepG2 and Huh7 cells were incubated with 100 ng/ml RANKL or PBS as control for 24 h. The number of HepG2 and Huh7 cells invading through the Transwell membrane increased significantly upon RANKL treatment (magnification ×200). ** *p*<0.01 compared with control.

To characterize the role of RANKL-RANK axis in the regulation of biological function of HCC cells, we first determined the expression of RANK by immunohistochemistry staining of the HCC tissue microarrays which contain 398 pairs of tumor and peritumor tissues. The results showed that RANK was obviously overexpressed in tumor tissues compared to the peritumor tissues in HCC. RANK staining was mainly observed in the cell membrane and cytoplasm of cancer cells (Figure 1A). Staining-intensity analysis demonstrated that percentage of HCC samples falling into grades − ∼ + was significantly lower in tumor tissues (15.33% & 50.50%) than in peritumor tissues (26.13% & 60.30%), whereas percentage of samples falling into grades ++ ∼ +++ was significantly higher in tumor tissues (16.33% &17.84%) than in peritumor tissues (11.56% &2.01%) (*p*<0.001; Figure 1A), which means a higher level of activated RANKL-RANK signaling pathway in tumor tissues than that in peritumor tissues.

We also analyzed the expression of RANK in Huh-7 and HepG2 HCC cell lines by qRT-PCR and Western blot analyses. A basal mRNA and protein expression of RANK were detected in both cells, which were increased significantly when the cells had been stimulated with RANKL for 24 hours (*p*<0.01; Figure 1B). Then, wound healing assay was performed to examine the role of RANKL in regulating the migration ability of Huh-7 and HepG2 cells. RANKL-stimulated Huh-7 and HepG2 cells rescued much more wounded region at 72 h after wound scratch compared with the relative untreated cells (*p*<0.01; Figure 1C). Further, the impact of RANKL on invasive capacity of Huh-7 and HepG2 cells was examined using Transwell invasion assay. Upon RANKL treatment, the number of Huh-7 and HepG2 cells invading through the Transwell membrane significantly increased compared to control groups (*p*<0.01; Figure 1D). Together, these results demonstrated that RANKL promoted migration and invasion of RANK-expressing HCC cells.

### RANKL modulated EMT-related molecules of HCC cells

**Figure 2:**
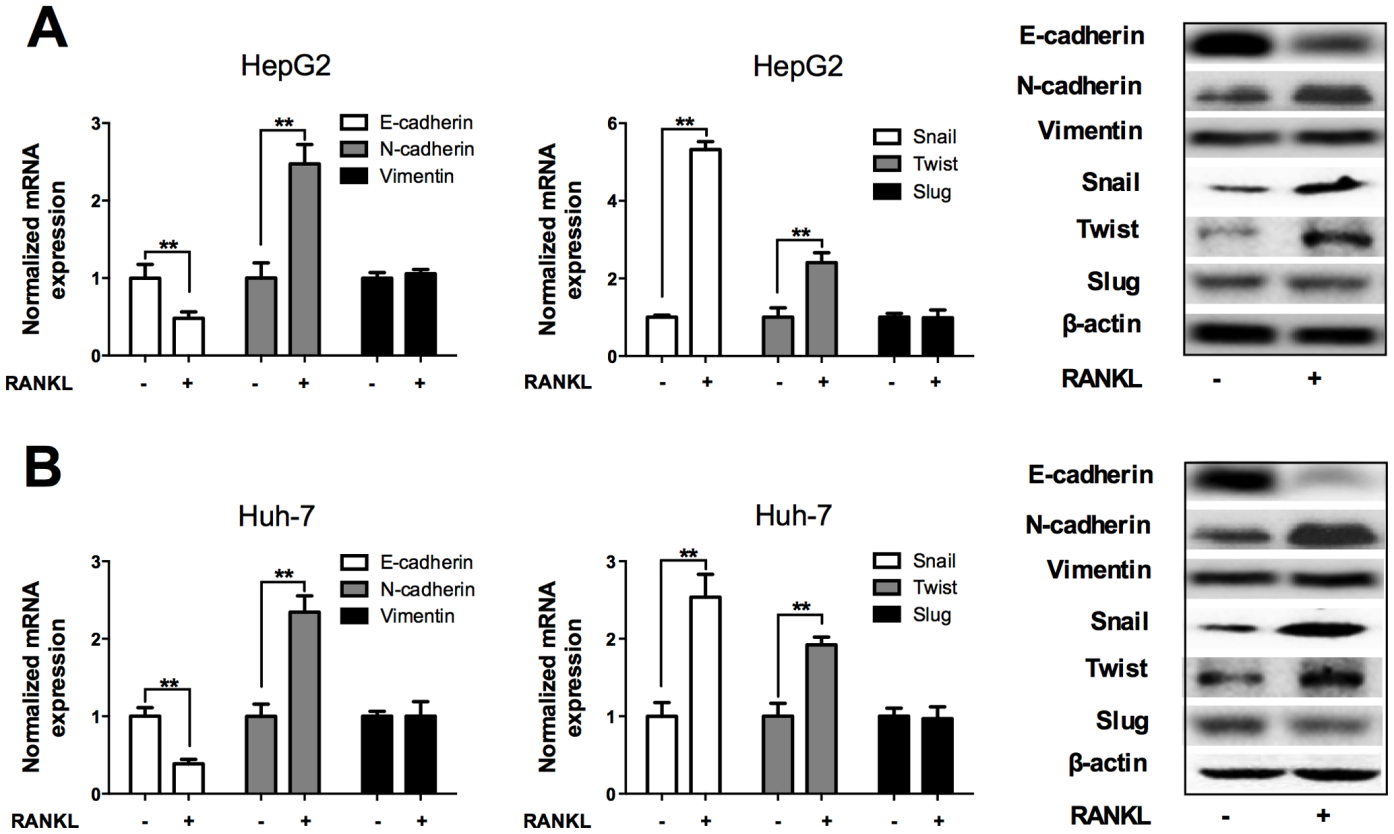
RANKL regulated the expression of EMT-related molecules in HCC cells. HepG2 and Huh7 cells were incubated with 100 ng/ml RANKL or PBS as control for 24 h. A. Both qRT-PCR and Western blot analyses revealed RANKL stimulation significantly promoted the expression of N-cadherin, Snail and Twist, inhibited E-cadherin, whereas the expression of vimentin and Slug did not show obvious changes. B. After treated with RANKL, Huh-7 showed a similar change in EMT markers as HepG2 cell. ** *p*<0.01 compared with control.

To investigate whether EMT contributes to the enhanced HCC cell migration and invasion induced by RANKL treatment, we examined the effect of RANKL on the expression of EMT markers in Huh-7 and HepG2 cells using qRT-PCR and Western blot analyses. In both cells, RANKL stimulation resulted in down-regulation of the epithelial marker E-cadherin, concomitant with up-regulation of the mesenchymal marker N-cadherin at both mRNA and protein levels, whereas the expression of vimentin had no obvious change (*p*<0.01; Figure 2A–B). Then we explored the effect of RANKL on the expression of EMT-related transcription factors in Huh-7 and HepG2 cells. RANKL stimulation significantly increased the mRNA and protein levels of Snail and Twist but not Slug in both cells (*p*<0.01; Figure 2A–B). Together, these data suggested that RANKL stimulation could confer HCC cells with EMT-like biochemical features, which might be responsible for RANKL-induced HCC cell migration and invasion.

### RANKL activated the NF-κB pathway in HCC cells

**Figure 3:**
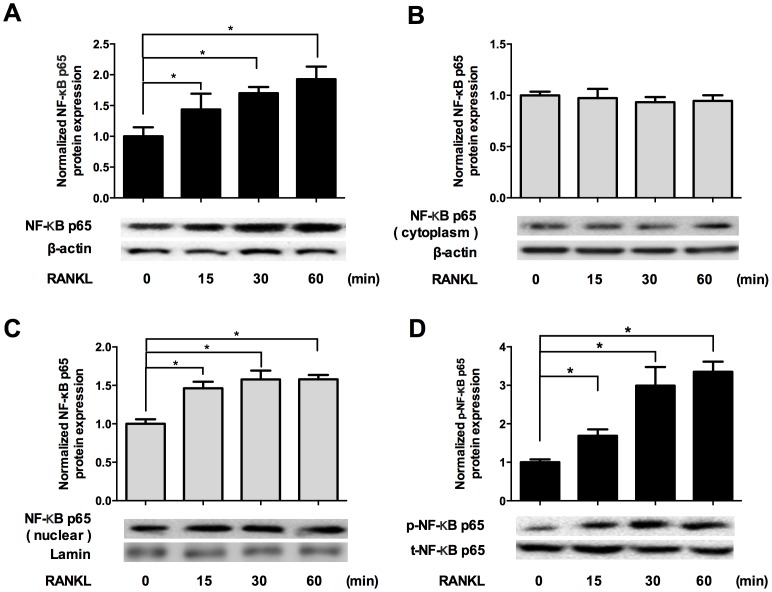
RANKL activated the NF-κB pathway in HCC cells. HepG2 cells were incubated with 100 ng/ml RANKL for 0, 15, 30 and 60 minutes. A. Western blot analysis indicated that total NF-κB p65 protein was up-regulated significantly upon RANKL treatment in a time-dependent manner. B. Treatment with RANKL did not change the expression of NF-κB p65 in cytoplasm as incubation time prolonged. C. RANKL stimulation significantly increased the expression of NF-κB p65 in the nucleus in a time-dependent manner. D. Western blot analysis revealed that phosphorylation of NF-κB p65 increased in a time-dependent manner upon RANKL treatment. * *p*<0.05 compared with 0 min group.

NF-κB signaling pathway has been implicated in RANKL/RANK-regulated cancer migration and invasion [20], [24].To investigate if RANKL could affect the activation of NF-κB pathway in HCC, Western blot analysis was performed to examine the total and subcellular levels of NF-κB p65 and the level of phosphorylated NF-κB p65 after treatment of HepG2 cells with RANKL for various time periods. The total level of NF-κB p65 protein was up-regulated upon RANKL treatment in a time-dependent manner (Figure 3A). Meanwhile, treatment with RANKL led to a time-dependent increase in the expression of NF-κB p65 in the nucleus but not cytoplasm (Figure 3B–C), indicating that RANKL could enhance p65 protein translocation into the nucleus, considering the up-regulation of total NF-κB p65 expression. We also found phosphorylated NF-κB p65 increased over time of after HCC cells were stimulated by RANKL (Figure 3D). Together, these results clearly showed that RANKL induced up-regulation, nuclear translocation, and phosphorylation of NF-κB p65 in HCC cells.

### NF-κB pathway was involved in RANKL-induced HCC cell migration, invasion, and EMT

**Figure 4:**
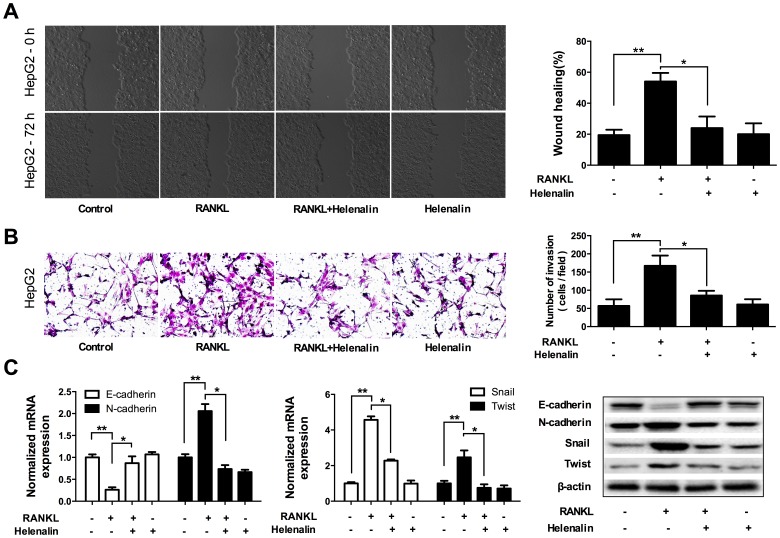
NF-κB pathway was involved in RANKL-induced migration, invasion and EMT of HCC cells. HepG2 cells were pretreated with 1 µM helenalin for 60 min, followed by incubating with 100 ng/ml RANKL for 24 h. A. Wound healing assay showed that pretreatment of HepG2 cells with helenalin significantly suppressed RANKL-induced cell migration. B. Transwell assay indicated helenalin abolished RANKL-induced invasion. C. qRT-PCR and Western blot showed that pretreatment with helenalin in HepG2 cells resulted in down-regulation of E-cadherin and up-regulation of N-cadherin, Snail, and Twist. ** *p*<0.01 compared with control, * *p*<0.05 compared with RANKL-treated group.

To identify if NF-κB signaling pathway is involved in the effects of RANKL on HCC cells, an NF-κB inhibitor, helenalin, was used. Pretreatment of HepG2 and Huh7 cells with helenalin significantly suppressed RANKL-induced cell migration, and invasion (*p*<0.01; Figures 4A–B and S1A–B). Moreover, helenalin pretreatment abolished RANKL-mediated down-regulation of E-cadherin and up-regulation of N-cadherin, Snail, and Twist (*p*<0.01; Figures 4C and S1C). Since MMP1, MMP3 and MMP9 were involved in tumor invasion and metastasis and can be regulated by NF-κB pathway [30], [31], [32], [33], we further investigated the effect of RANKL on the MMPs. Western blot indicated stimulation by RANKL would promote the expression of MMP1, MMP3 and MMP9 in both HepG2 and Huh7 cell line (Figure S2). Together, these data indicated that NF-κB pathway contributed to RANKL-induced motility, invasion and EMT of HCC cells.

## Discussion

RANKL-RANK axis is essential for normal physiological processes like immune responses and bone remodeling [18].Notably, RANKL-RANK axis also plays an important role in invasion and metastasis of various human cancers [19]. However, little is known about the role of this axis in human HCC. Herein, we showed that RANK, the receptor of RANKL, was significantly up-regulated in HCC tumor tissues compared with paired peritumor liver tissues. The expression of RANK in HCC cells can be up-regulated upon RANKL stimulation. Further, *in vitro* experiments showed that activation of RANKL-RANK axis significantly potentiated HCC cell migration and invasion ability by promoting EMT. Moreover, RANKL-induced EMT in HCC cells was completed through the up-regulation of Snail and Twist which depended on activation of NF-κB signaling. The results demonstrated a causal role of RANKL-RANK axis in HCC cell migration and invasion.

Previous studies have shown that RANKL and RANK are expressed in HCC, and RANKL expression correlates with poor clinical outcome [28], [34], [35]. However, how many percentage of HCC patients showed an up-regualtion of RANK and the exact role of RANKL-RANK axis in HCC remains elusive. Here, immunohistochemical study in a large cohort of 398 HCC patients revealed that RANK expression was significantly higher in tumor tissues than in peritumor tissues. Percentage of HCC samples falling into grades − ∼ + was significantly lower in tumor tissues (15.33% & 50.50%) than in peritumor tissues (26.13% & 60.30%), whereas percentage of samples falling into grades ++ ∼ +++ was significantly higher in tumor tissues (16.33% &17.84%) than in peritumor tissues (11.56% &2.01%). Furthermore, our *in vitro* experiments showed that RANKL stimulation could markedly increase migratory and invasive ability of HCC cells, with obvious down-regulation of the epithelial marker E-cadherin and up-regulation of the mesenchymal marker N-cadherin. Transcription factors, such as Snail, Slug, and Twist, are pivotal activators of EMT [10]. Snail and Twist have been reported to mediate EMT, resulting in tumor progression, and poor survival in patients with HCC. Slug, however, did not seem to affect the outcome. Our results showed that RANKL stimulation led to significant increases in the expression of Snail and Twist, but not Slug. Despite lack of direct evidence, our findings strongly suggested that RANKL induced EMT through up-regulation of Snail and Twist, thereby favoring HCC cell migration and invasion. Moreover, we found that RANKL could promote the expression of MMP1, MMP3 and MMP9, which indicated that increased expression of MMPs was involved in the process that RANKL promoted tumor invasion and migration via NF-κB pathway. Collectively, these findings suggest that RANKL play a key role in the process of HCC metastasis.

Meanwhile, our results showed that RANKL stimulation induced up-regulation, nuclear translocation, and phosphorylation of the NF-κB p65, indicating activation of the NF-κB signaling pathway. Moreover, pharmacological inhibition of NF-κB pathway attenuated RANKL-induced motility, invasion and EMT of HCC cells. Therefore, it was reasonable to assume that NF-κB was involved in RANKL-dependent migration, invasion and EMT effects on HCC cells.

In conclusion, we demonstrated that RANK was significantly up-regulated in human HCC, and RANKL stimulation can lead directly to migration, invasion, and EMT of HCC cells via NF-κB signaling. Therefore, RANKL-RANK axis may have critical role in regulating the metastatic potential of HCC cells, providing an attractive therapeutic target for HCC treatment.

## Supporting Information

Figure S1 **NF-κB pathway was involved in RANKL-induced migration, invasion and EMT of Huh7 cells.** Huh7 cells were pretreated with 1 µM helenalin for 60 min, followed by incubating with 100 ng/ml RANKL for 24 h. A. Wound healing assay showed that pretreatment of Huh7 cells with helenalin significantly suppressed RANKL-induced cell migration. B. Transwell assay indicated helenalin abolished RANKL-induced invasion. C. qRT-PCR and Western blot showed that pretreatment with helenalin in Huh7 cells resulted in down-regulation of E-cadherin and up-regulation of N-cadherin, Snail, and Twist. ** *p*<0.01 compared with control, * *p*<0.05 compared with RANKL-treated group.
(DOCX)

Figure S2 **Stimulation by RANKL promoted the expression of MMPs in HCC cell lines.** HepG2 and Huh7 cells were incubated with 100 ng/ml RANKL or PBS as control for 24 h. Western blot indicated RANKL promoted the expression of MMP1, MMP3 and MMP9 compared to the control group.
(DOCX)

Table S1 **Sequence of primers for qRT-PCR.**
(DOCX)

Table S2 **Primary antibodies for Western blot and immunohistochemistry.**
(DOCX)
