# Supplementary figures and images for: RANKL Promotes Migration and Invasion of Hepatocellular Carcinoma Cells via NF-κB-Mediated Epithelial-Mesenchymal Transition
Source: PLoS One. 2014 Sep 30;9(9):e108507. doi: 10.1371/journal.pone.0108507 (PMC4182493; doi:10.1371/journal.pone.0108507)

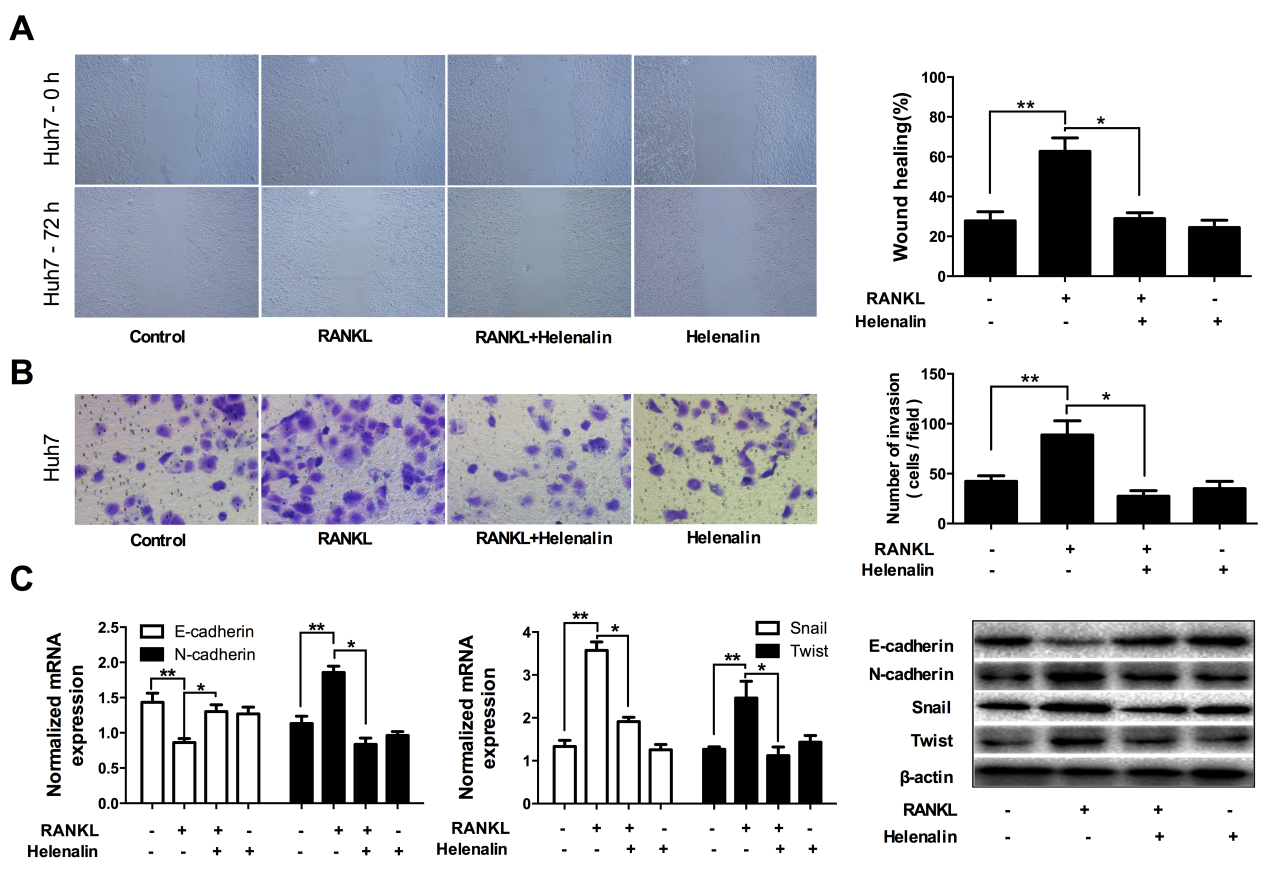

Supplement: Figure S1 — NF-κB pathway was involved in RANKL-induced migration, invasion and EMT of Huh7 cells. Huh7 cells were pretreated with 1 µM helenalin for 60 min, followed by incubating with 100 ng/ml RANKL for 24 h. A. Wound healing assay showed that pretreatment of Huh7 cells with helenalin significantly suppressed RANKL-induced cell migration. B. Transwell assay indicated helenalin abolished RANKL-induced invasion. C. qRT-PCR and Western blot showed that pretreatment with helenalin in Huh7 cells resulted in down-regulation of E-cadherin and up-regulation of N-cadherin, Snail, and Twist. ** p<0.01 compared with control, * p<0.05 compared with RANKL-treated group. (DOCX) [file pone.0108507.s001.docx]

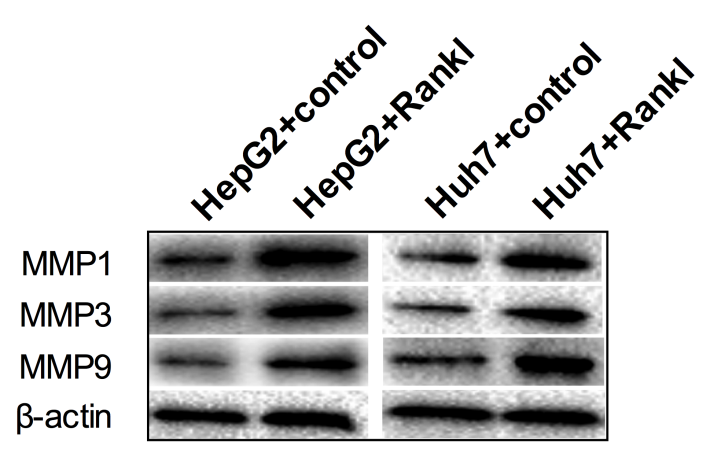

Supplement: Figure S2 — Stimulation by RANKL promoted the expression of MMPs in HCC cell lines. HepG2 and Huh7 cells were incubated with 100 ng/ml RANKL or PBS as control for 24 h. Western blot indicated RANKL promoted the expression of MMP1, MMP3 and MMP9 compared to the control group. (DOCX) [file pone.0108507.s002.docx]
