# Supplementary material for: RANKL Promotes Migration and Invasion of Hepatocellular Carcinoma Cells via NF-κB-Mediated Epithelial-Mesenchymal Transition
Source: PLoS One. 2014 Sep 30;9(9):e108507. doi: 10.1371/journal.pone.0108507 (PMC4182493; doi:10.1371/journal.pone.0108507)
Supplement: Table S1 — Sequence of primers for qRT-PCR. (DOCX) [file pone.0108507.s003.docx]

| **Gene** |  | **Primer Sequence** |
| --- | --- | --- |
| RANK |  | F: 5'-TTGTGGCACTGGATCAATGAGG-3' |
|  |  | R: 5'-CCGTGAAGCACTGGCTTAAACTG-3' |
| E-Cadherin |  | F: 5'-ATTGCTCACATTTCCCAACTCC-3' |
|  |  | R: 5'-CTCTGTCACCTTCAGCCATCCT-3' |
| N-Cadherin |  | F: 5'-CAGACATGGAAGGCAATCCCACA-3' |
|  |  | R: 5'-CTGGATGGCGAACCGTCCAGTAGGA-3' |
| vimentin |  | F: 5'-GCTGAATGACCGCTTCGCCAACT-3' |
|  |  | R: 5'-GCTCCCGCATCTCCTCCTCGTA-3' |
| Snail |  | F: 5'-TTTACCTTCCAGCAGCCCTACGA-3' |
|  |  | R: 5'-GGAGCCTTTCCCACTGTCCTCAT-3' |
| Slug |  | F: 5'-TTCCGATCAGCCTGCCTTTAGA-3' |
|  |  | R: 5'-TTTGCCTTGCACAAAGACCAAA-3' |
| Twist |  | F: 5'-GCCGACGACAGCCTGAGCAACA-3' |
|  |  | R: 5'-CGCCACAGCCCGCAGACTTCTT-3' |
| GAPDH |  | F: 5'-GAGTCAACGGATTTGGTCGTATTG-3' |
|  |  | R: 5'-CCTGGAAGATGGTGATGGGATT-3' |
