# Supplementary material for: RANKL Promotes Migration and Invasion of Hepatocellular Carcinoma Cells via NF-κB-Mediated Epithelial-Mesenchymal Transition
Source: PLoS One. 2014 Sep 30;9(9):e108507. doi: 10.1371/journal.pone.0108507 (PMC4182493; doi:10.1371/journal.pone.0108507)
Supplement: Table S2 — Primary antibodies for Western blot and immunohistochemistry. (DOCX) [file pone.0108507.s004.docx]

| **Antibodies** | **Concentration for WB** |  | **Concentration for IHC** |  | **Specificity** | **Company** |
| --- | --- | --- | --- | --- | --- | --- |
| RANK | 1/500 |  | 1:100 |  | Mouse polyclonal | Abcam |
| E-Cadherin | 1/1000 |  | - |  | Rabbit polyclonal | Cell Signaling |
| N-Cadherin | 1/1000 |  | - |  | Rabbit polyclonal | Cell Signaling |
| vimentin | 1/1000 |  | - |  | Rabbit polyclonal | Cell Signaling |
| Snail | 1/1000 |  | - |  | Rabbit polyclonal | Cell Signaling |
| Slug | 1/1000 |  | - |  | Rabbit polyclonal | Cell Signaling |
| Twist | 1/400 |  | - |  | Rabbit polyclonal | Abcam |
| NF-κB p65 | 1/1000 |  | - |  | Rabbit polyclonal | Cell Signaling |
| Phospho-NF-κB p65 | 1/1000 |  | - |  | Rabbit polyclonal | Cell Signaling |
| MMP1 | 1/500 |  |  |  | Rabbit polyclonal | Abcam |
| MMP3 | 1/500 |  |  |  | Rabbit polyclonal | Abcam |
| MMP9 | 1/500 |  |  |  | Rabbit polyclonal | Abcam |
| Actin | 1/1000 |  | - |  | Rabbit polyclonal | Abcam |
| Lamin B1 | 1/1000 |  | - |  | Rabbit polyclonal | Abcam |
